# Sex‐specific associations between traumatic experiences and resting‐state functional connectivity in the Philadelphia Neurodevelopmental Cohort

**DOI:** 10.1002/jcv2.12049

**Published:** 2021-12-06

**Authors:** Shiying Wang, Jeffrey G. Malins, Heping Zhang, Jeffrey R. Gruen

**Affiliations:** ^1^ Department of Biostatistics Yale University School of Public Health New Haven Connecticut USA; ^2^ Department of Psychology Georgia State University Atlanta Georgia USA; ^3^ Haskins Laboratories New Haven Connecticut USA; ^4^ Departments of Pediatrics and Genetics Yale University School of Medicine New Haven Connecticut USA

**Keywords:** default mode network, functional connectivity, sex differences, somatomotor network, traumatic experiences

## Abstract

**Background:**

Traumatic experiences during childhood or adolescence are a significant risk factor for multiple psychiatric disorders and adversely affect multiple cognitive functions. Resting‐state functional magnetic resonance imaging has been used to investigate the effects of traumatic experiences on functional connectivity, but the impact of sex differences has not been well documented. This study investigated sex‐specific associations between resting‐state functional connectivity (rsFC) and traumatic experiences in typically developing youth.

**Methods:**

The sample comprised 1395 participants, aged 8–21 years, from the Philadelphia Neurodevelopmental Cohort. Traumatic experiences were assessed based on the structured psychiatric evaluation. Sex, the number of traumatic events, and their interaction were regressed onto voxel‐wise intrinsic connectivity distribution parameter values derived from resting‐state functional magnetic resonance imaging. Brain regions that passed cluster correction were used as seeds to define resting‐state networks.

**Results:**

After quality control, the final sample had 914 participants with mean (*SD*) age 14.6 (3.3) years; 529 (57.8%) females; 437 (47.8%) experienced at least one kind of traumatic event. Four discrete anatomical clusters showed decreased functional connectivity as the number of traumatic events increased. The resting‐state networks defined by using these four clusters as seeds corresponded with the somatomotor network. Sex‐specific associations were identified in another three clusters for which males showed increased connectivity, and females showed decreased connectivity as the number of traumatic events increased. The resting‐state networks defined by the three sex‐specific clusters corresponded with the default mode network (DMN).

**Conclusions:**

In youth without psychiatric diagnoses, traumatic experiences are associated with an alteration of rsFC in brain regions corresponding with the somatomotor network. Associations differ in direction between males and females in brain regions corresponding with the DMN, suggesting sex‐specific responses to early exposure to trauma.

## Graphical abstract

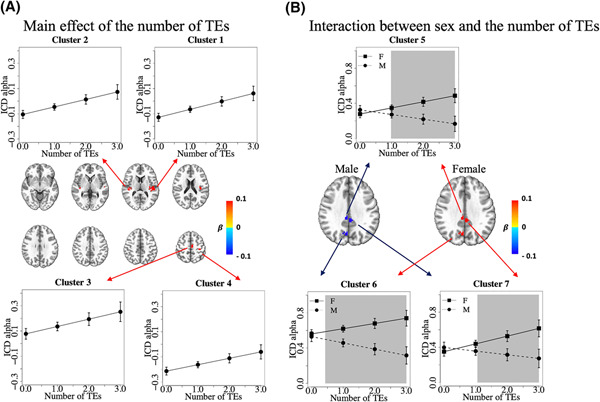

Little is known about the sex‐specific effects of traumatic experiences on functional connectivity. In a large sample of non‐psychiatric youth (914 participants from the Philadelphia Neurodevelopmental Cohort), we show an association between traumatic experiences and reduced resting‐state functional connectivity in brain regions corresponding with the somatomotor network (Figure 1(a)). In Figure 1(b), we show sex‐specific associations between traumatic experiences and resting‐state functional connectivity in brain regions corresponding with the default mode network; for males, resting‐state functional connectivity increased (ICD alpha value decreased) whereas for females resting‐state functional connectivity decreased (ICD alpha value increased) as exposure to the number of traumatic events increased.

**Key points**

- Traumatic experiences are a significant risk factor for multiple psychiatric disorders and adversely affect multiple cognitive functions. Little is known about the sex‐specific effects of traumatic experiences on functional connectivity
- In a large sample of non‐psychiatric youth, this study identified an association between traumatic experiences and reduced resting‐state functional connectivity (rsFC) in brain regions corresponding with the somatomotor network
- This study also identified sex‐specific associations between traumatic experiences and rsFC in brain regions corresponding with the default mode network
- The current findings motivate future research to further clarify brain linkages between experiencing traumatic events in childhood and long‐standing effects on language and somatosensory processing, as well as differences in intrusive memory recall and risk of post‐traumatic stress disorder between the sexes

## INTRODUCTION

Traumatic experiences during childhood or adolescence, defined as exposure to actual or threatened death, serious injury, or sexual abuse (American Psychiatric Association, 2013), are a significant risk factor for multiple psychiatric disorders, such as post‐traumatic stress disorder (PTSD) and major depressive disorder (Copeland et al., 2007; Kilpatrick et al., 2003). Traumatic experiences are associated with adverse effects on multiple cognitive functions including processing speed, memory, and language skills (Majer et al., 2010; Sylvestre et al., 2016). In the United States, the prevalence of traumatic experiences during childhood and adolescence ranges from 10.14% to 23.3% (Briere & Elliott, 2003; Pérez‐Fuentes et al., 2013). Furthermore, sex differences in trauma‐related psychopathology have been documented. For example, females are more likely than males to develop PTSD after experiencing trauma (Tolin & Foa, 2008). However, the biological mechanisms underlying sex‐specific associations between traumatic experiences and psychopathology are not well understood.

Functional magnetic resonance image (fMRI) is a powerful tool for analyzing the neurobiology of cognitive functioning. In particular, resting‐state fMRI can delineate intrinsic functional brain networks enabling auditory and visual processing, attention, memory retrieval, and many of the critical functions collectively called cognition. Resting‐state functional connectivity (rsFC), which examines inter‐correlations of activity between brain regions, has been utilized to investigate the impact of trauma on brain function. Compared to controls, PTSD is associated with decreased rsFC within the default mode network (DMN; Bluhm et al., 2009; Sripada et al., 2012; Viard et al., 2019), increased rsFC within the salience network, and elevated cross‐network connectivity between both networks (Sripada et al., 2012). Traumatic exposure in individuals without PTSD is associated with alterations within the DMN (Lu et al., 2017) and insula‐based networks (Marusak et al., 2015). These findings suggest that traumatic experiences play a central role in rsFC changes in individuals with and without PTSD. However, the sample sizes of these association studies are small and lack power to stratify for sex and other covariates.

We hypothesize that associations between traumatic exposure and rsFC differ between the sexes. This hypothesis is partly predicated on sex differences in neural activation for PTSD. In an fMRI fear‐perception paradigm, when compared to males, females with PTSD showed greater activity in the dorsal brainstem and less activity in the hippocampus (Felmingham et al., 2010). Furthermore, sex differences in rsFC have been observed in emotional processing for healthy adults (Wu et al., 2016) and adolescents (Alarcón et al., 2015). Therefore, we hypothesize there are sex‐specific associations between traumatic experience and rsFC. Specifically, females and males may show different patterns of rsFC in particular brain regions as the number of traumatic experiences increases.

We tested this hypothesis in the Philadelphia Neurodevelopmental Cohort (PNC; Satterthwaite et al., 2016; Hakonarson & Gur, 2017), a large non‐psychiatric sample of adolescents. The large sample size provided significant power for detecting low signal from background noise inherent to fMRI studies, as well as stratification by sex and correction for age and socioeconomic factors. Studies analyzing the PNC have reported sex differences in functional connectivity (Satterthwaite et al., 2015) and altered rsFC associated with PTSD symptoms and trauma exposure (Sheynin et al., 2020). However, sex‐specific associations between traumatic exposure and rsFC have not been inspected in this cohort.

## MATERIALS AND METHODS

### Participants

The PNC (Satterthwaite et al., 2016) is a large non‐psychiatric sample of youth aged 8–21 years. Data were collected through a collaborative study between the Center for Applied Genomics at Children’s Hospital of Philadelphia and the Brain Behavior Laboratory at the University of Pennsylvania from 2009 to 2011. For this study, we analyzed a subsample of 1395 participants with both structured psychiatric evaluations and resting‐state fMRI brain scans.

### Traumatic experience assessment

Each participant received a structured screening interview, called GOASSESS (Calkins et al., 2014). We used nine questions from the PTSD assessment scale (Table S1) to assess traumatic experiences (Barzilay et al., 2019). Responses for each question were assigned a value of 1 for yes, and 0 for no (Appendix S1). For each participant, we summed the number of unweighted traumatic events (TEs). The number of participants who experienced three or more TEs was relatively small, which might result in unstable estimation of effect size in the regression analysis. For this reason, we grouped these participants into one group, and the number of TEs was coded as 0, 1, 2, or 3 for use in subsequent regression analyses.

### Image acquisition and preprocessing of MRI data

MRI images were collected using a single 3T Siemens TIM Trio whole‐body scanner located in the Hospital of the University of Pennsylvania (Satterthwaite et al., 2016). The acquisition of anatomical images and resting‐state fMRI are detailed in Appendix S2. The total duration of the resting‐state scan was 6.2 min. We preprocessed resting‐state fMRI data in AFNI (Cox, 1996). The first four volumes (repetition times, or TRs) were removed, followed by slice‐scan time correction, alignment with anatomic images, and registration to MNI152 space. We corrected for head motion by aligning to the volume with the minimum outlier fraction, smoothed images using a 6 mm FWHM Gaussian kernel, and performed a general linear model using six motion parameters and their derivatives as regressors, as well as frequency components between 0.01 and 0.08 Hz. Volumes were censored if they exceeded 0.3 mm point‐to‐point Euclidean movement and/or had >10% outlier voxels (Appendix S3). The pre‐processing procedure was similar to Jalbrzikowski et al. (2020), whereby resting‐state fMRI data from the PNC were pre‐processed with 12 motion parameters, and participants were removed if they had greater than 30% of volumes with a framewise displacement greater than 0.3 mm. Then, we performed tissue‐based regression to regress out the average signals of individual eroded white matter masks and the first three principal components of individual eroded lateral ventricle masks (Appendix S4).

### Quality control

After preprocessing of fMRI data, we removed participants with average motion per TR > 0.3 mm or censor fractions >30% (*n* = 225). Participants whose functional and anatomic images did not co‐register properly (based on visual inspection) were removed (*n* = 69). Based on self‐reported medical history, participants with serious brain injury were excluded (*n* = 178). In addition, two participants were excluded for missing traumatic experience records, and seven were excluded for missing data concerning maternal education. Data from 914 participants passed quality control procedures and were used in the primary analyses. The average scan duration post‐QC in the included participants was 5.68 min. Demographic information and the number of TEs for included and excluded participants are presented in Table S2.

### Intrinsic connectivity distribution (ICD)

We used ICD to measure voxel‐level functional connectivity (Scheinost et al., 2012). ICD is a data‐driven method that does not require prior information to define regions of interest or an arbitrarily chosen correlation threshold. For each voxel, a histogram of positive correlation coefficients was generated to characterize the rsFC between that voxel and all other voxels. These histograms were approximated using a function with two parameters, alpha and beta (Appendix S5). Alpha describes the variance, whereas beta describes the decay rate, such that a smaller alpha and a larger beta for any voxel represent a relatively larger amount of strong connections and higher connectivity with other voxels. We computed ICD parameter values for voxels within an MNI152 gray matter mask (31,053 voxels) for each participant using custom scripts in R (version 3.6.0). The R script is publicly available at https://github.com/ShiyingWang1014/Intrinsic‐connectivity‐distribution.

### Regression on ICD parameter values

We conducted regression analyses on subject‐wise ICD parameter values to define seeds for functional connectivity analysis. ICD parameter values were smoothed using a 4 mm FWHM Gaussian kernel. In the regression model for each voxel, ICD parameter values were regressed on the number of TEs that participants experienced. Covariates included sex, age, and maternal education (measured in years). To test for sex‐specific associations, interaction terms between sex and the number of TEs were also included in the model. ICD alpha and beta values were respectively analyzed. Cluster correction was performed with the voxel‐wise threshold set at *p* = 0.001, cluster corrected at *α* = 0.05.

### Seed‐based functional connectivity analysis

We computed correlation maps using the clusters that passed correction as seed regions. Correlation coefficients between the time series of a seed region and each voxel were calculated and transformed to *z* scores. Then, using a *t*‐test, we tested whether *z* scores were significantly different from 0. Regions passing the thresholds of *p* < 1 × 10^−44^ and FDR < 3 × 10^−16^ were defined as resting‐state networks. Similar to the method of Huang and Barber (2021) in the same cohort, we used stringent thresholds because the extent of tissue that surpassed the significance threshold at lower levels (such as FDR < 0.01) was too large to detect circumscribed clusters. Correlation maps were drawn using SUMA for visualization purposes (Saad et al., 2004). Identified networks (volume‐based) were compared with seven well‐defined reference networks (Thomas Yeo et al., 2011) to determine the percentage of voxels overlapping with each reference network. The parcellation of reference networks was downloaded from https://surfer.nmr.mgh.harvard.edu/fswiki/CorticalParcellation_Yeo2011.

## RESULTS

### Demographics

After quality control, the final sample had 914 participants with mean (*SD*) age 14.6 (3.3) years; 529 (57.8%) were females, 423 (46.3%) were African American, 396 (43.3%) were white, and 437 (47.8%) experienced at least one kind of TE. Table S3 shows the demographic information for participants by the number of TEs. Participants who experienced more TEs were older and had fewer years of maternal education. The demographic information of participants with and without traumatic exposure, as well as the frequency of participants in each category of TE, are provided in Tables S4 and S5.

### Regression on ICD parameter values

Using resting‐state fMRI data, we computed ICD parameter values for voxels within a gray matter mask for each participant. Then, we conducted a regression analysis on ICD parameter values to analyze the main effect of the number of TEs and the interaction between sex and the number of TEs. No clusters were significant for the beta parameter after cluster correction. Results reported here are for the alpha parameter.

**FIGURE 1:**
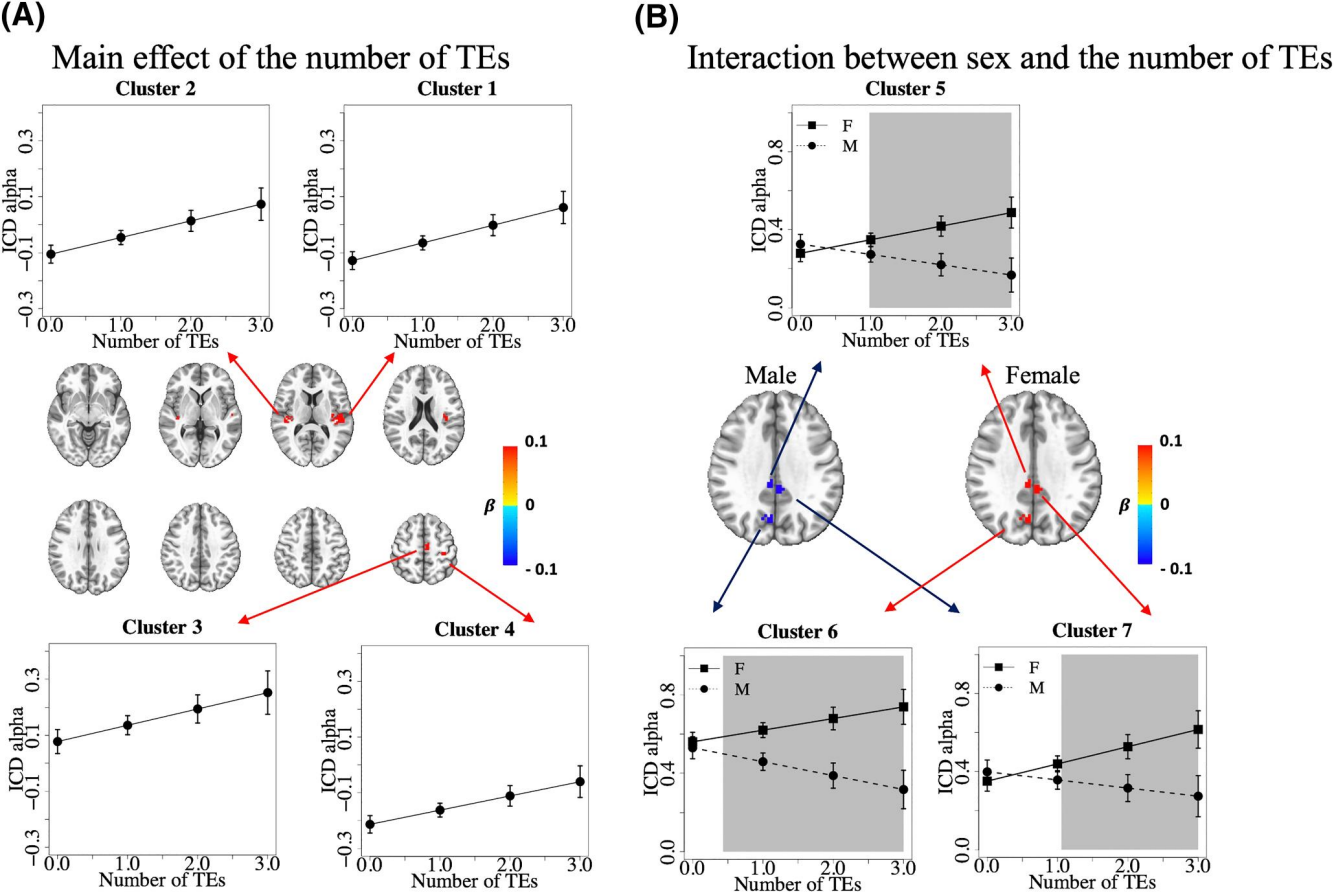
Significant clusters that passed cluster correction in regression on intrinsic connectivity distribution (ICD) alpha values. (A) Significant clusters for the main effect of the number of traumatic events (TEs) on ICD alpha values. Four clusters passed cluster correction (voxelwise *p* < 0.001, cluster corrected at *p* < 0.05). Colors represent the value of the regression coefficient β for the number of TEs for voxels within significant clusters. The space between layers is 9 mm. Linear regression plots show the average ICD alpha value of voxels within the cluster versus the number of TEs. (B) Significant clusters for the interaction between sex and the number of traumatic events. Three clusters passed cluster correction (voxelwise *p* < 0.001, cluster corrected at *p* < 0.05). To better visualize divergent effect directions for females and males in these clusters, the regression coefficients for the main effect term (the number of TEs) and the interaction term (sex × the number of TEs) in the regression model were integrated. Colors represent the amount of ICD alpha value change as the number of TEs increased one unit for males and females. Linear regression plots show the average ICD alpha value of voxels within the cluster versus the number of TEs by sex. The shaded gray boxes indicate regions where the confidence intervals of points on the regression lines for males and females do not overlap (i.e., regions where the estimated ICD alpha values are significantly different by sex). A smaller ICD alpha represents a higher connectivity with other voxels. Anatomical regions corresponding to the clusters are listed in Table 1

After cluster correction, we identified four clusters that showed a significant main effect of the number of TEs on ICD alpha values (Table 1, Clusters 1–4). Within these clusters, as the number of TEs increased, alpha values increased (Figure 1A), which means that among participants with higher TE exposure, voxels in these regions had reduced functional connectivity with other voxels. All four clusters retained significance after removing the interaction between sex and the number of TEs (Table S6). Except for Cluster 3, Clusters 1, 2, and 4 retained significance after removing all covariates from the model (Table S7).

**TABLE 1:** Summary of significant clusters that passed cluster correction in regression on ICD alpha values

Cluster^a^	Size (voxels)	Hemisphere	Peak voxel^b^	Average effect size^c^	Regions (% of each region in cluster)
Significant clusters for the main effect of the number of TEs on ICD alpha values					
1	106	Left	(−52.5, −22.5, 7.5)	0.066	Superior temporal gyrus (49.1), Heschl’s gyrus (20.8), Rolandic operculum (16.9), and insula (12.9)
2	49	Right	(37.5, −25.5, 13.5)	0.062	Heschl’s gyrus (46.3), insula (20.3), and superior temporal gyrus (19.7)
3	24	Left	(−4.5, −13.5, 55.5)	0.067	SMA (49.4) and paracentral lobule (48.5)
4	20	Left	(−31.5, −28.5, 55.5)	0.053	Precentral gyrus (60.5) and postcentral gyrus (39.5)
Significant clusters for the interaction between sex and the number of TEs on ICD alpha values					
5	37	Right	(7.5, −46.5, 13.5)	−0.12	Posterior cingulate cortex (40.4), middle cingulate cortex (30.2), and precuneus (25.9)
6	29	Right	(7.5, −67.5, 34.5)	−0.13	Precuneus (85.6) and cuneus (12.4)
7	22	Left	(−4.5, −37.5, 34.5)	−0.13	Posterior cingulate cortex (66.6) and middle cingulate cortex (31.4)

Abbreviations: ICD, intrinsic connectivity distribution; SMA, supplementary motor area; TE, traumatic event.

^a^ Clusters 1 and 2 have cluster corrected *p* << 0.01, Clusters 5 and 6 have cluster corrected *p* < 0.01, Clusters 3 and 7 have a cluster corrected *p* < 0.02, and Cluster 4 has a cluster corrected *p* < 0.03.

^b^ Peak voxel: The coordinate of the voxel with the largest absolute regression coefficient value within each cluster in MNI152 space in LPI orientation.

^c^ Average effect size: the average effect size of voxels within the cluster. For the main effect of the number of TEs, the effect size represents the change of alpha value as the number of TEs increases by 1. For the interaction between sex and the number of TEs, the effect size represents the difference between males and females in the change of alpha values as the number of TEs increases by 1.

We identified three additional clusters that showed a significant interaction between sex and the number of TEs on ICD alpha values (Table 1, Clusters 5–7). Within these clusters, males and females had divergent changes in rsFC. For males, rsFC increased (ICD alpha value decreased) whereas for females rsFC decreased (ICD alpha value increased) as the number of TEs increased (Figure 1B). All three clusters retained significance after removing all covariates from the model (Table S7). Furthermore, regressing ICD values on age alone, or on age, maternal education, sex, number of TEs, and the interaction between sex and the number TEs, revealed that regions showing significant age‐related effects were distinct from those showing a main effect of the number of TEs or the interaction between sex and the number of TEs (Figures S1 and S2).

### Seed‐based functional connectivity analysis

To identify correspondences between clusters and resting‐state networks, we used all seven clusters as seed regions to construct correlation maps and to define seven resting‐state networks.

**FIGURE 2:**
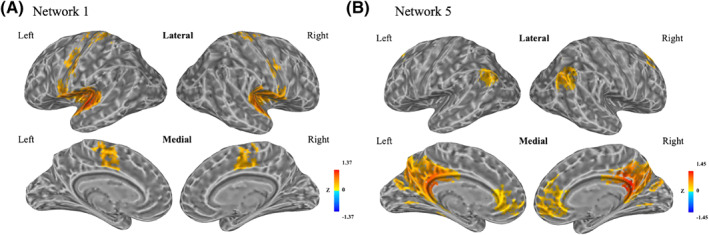
Resting‐state Network 1 and resting‐state Network 5. (A) Resting‐state Network 1 defined by using Cluster 1 as a seed region. (B) Resting‐state Network 5 defined by using Cluster 5 as a seed region. Correlation coefficients between the average time‐series data of the seed region with every other voxel were calculated and transformed to z scores. A *t*‐test identified significant regions (*p* < 1 × 10^−44^, FDR < 3 × 10^−16^) that defined a resting‐state network. Colors represent the value of *z* scores within defined resting‐state networks

Using Cluster 1 as a seed region, we constructed resting‐state Network 1. It contained bilateral superior temporal gyri, Heschl’s gyri, supplementary motor area (SMA), middle cingulate cortex, precentral gyri, postcentral gyri, Rolandic operculum and insula, and the left paracentral lobule (Figures 2A and S3). We compared Network 1 with seven well‐defined resting‐state networks (Thomas Yeo et al., 2011). Among the total of 1722 voxels in Network 1, 82.5% overlapped with the somatomotor network (Table S8), which included somatomotor and auditory cortices. Network 1 included most of the cortical regions in Networks 2–4. The percentages of Networks 2–4 overlapping with Network 1 were 98.4%, 58.2%, and 56.4%, respectively (Figures S4–S6 and Tables S9–S11).

Using Cluster 5 as a seed region, we constructed resting‐state Network 5. It contained bilateral medial‐orbital gyri, anterior cingulate cortices, angular gyri, precuneus, middle cingulate cortices, posterior cingulate cortices (PCC), middle frontal gyri and cuneus, the left middle occipital gyrus, and the right superior frontal gyrus (Figure 2B and Figure S7). We compared Network 5 with seven reference resting‐state networks (Thomas Yeo et al., 2011). Among 1737 voxels in Network 5, 80.1% overlapped with the DMN (Table S8). Network 5 included most of the cortical regions in Networks 6 and 7. The percentages of Networks 6 and 7 overlapping with Network 5 were 79% and 95.9%, respectively (Figures S8 and S9 and Tables S12 and S13).

## DISCUSSION

In a large cohort of non‐psychiatric youth, we investigated sex‐specific associations between traumatic experiences and rsFC. We identified four anatomical clusters that showed decreased rsFC with other brain regions as the number of TEs increased. The defined resting‐state networks using these clusters as seeds corresponded with the somatomotor network. We identified another three brain clusters that showed an effect of the number of TEs, but in opposite directions for males and females. The defined resting‐state networks using these clusters as seeds highly corresponded with the DMN.

### Main effect of the number of TEs

The brain regions that showed significant associations between traumatic experiences and rsFC are similar to those identified in previous studies of healthy adults with traumatic exposure (Lu et al., 2017; Philip, Kuras, et al., 2013) and patients with PTSD (Tursich et al., 2015; Zhang et al., 2016).

Clusters 1 and 2 were mainly centered on temporal gyri, including the posterior temporal gyrus and Heschl’s gyrus. Temporal gyri play an essential role in auditory processing for receptive and expressive language. Wernicke’s area, located in the posterior portion of the left superior temporal gyrus, is critical for speech processing and language comprehension. In a small study of 48 young adults (average age 21.8 years), decreased regional homogeneity was observed in the bilateral posterior temporal gyri among participants with childhood trauma (Lu et al., 2017). Decreased regional homogeneity in the right posterior temporal gyrus was also reported in a small study of healthy adults (Philip, Kuras, et al., 2013). Tursich et al. (2015) studied 21 adults with PTSD related to childhood trauma, and observed an association between decreased rsFC of the left posterior temporal gyrus and more severe hyperarousal symptoms. Behaviorally, children who had experienced maltreatment were found to have delayed language skills, which are associated with temporal gyri, compared to children without maltreatment experiences (Sylvestre et al., 2016).

Clusters 1 and 2 also contained the posterior insula. The insular cortex subserves different functions, including sensorimotor processing, emotional processing, and cognitive functioning (Uddin et al., 2017). The posterior insula has been functionally associated with primary and secondary somatomotor cortices, including the SMA, pre‐SMA, and most of the precentral and postcentral gyri (Deen et al., 2011). Zhang et al. (2016) observed that relative to controls (*n* = 20), patients with PTSD (*n* = 20) who experienced serious vehicle accidents showed decreased rsFC between the right posterior insula and the postcentral gyrus. These reported results are consistent with our current finding that the right posterior insula and postcentral gyrus exhibited decreased functional connectivity as the number of TEs increased.

Clusters 3 and 4 included sensorimotor regions such as the left SMA, paracentral lobule, and precentral and postcentral gyri. The disruption of rsFC in these sensorimotor regions has not been reported in non‐psychiatric individuals with traumatic experiences. For patients with PTSD, Zhang et al. (2016) observed decreased functional connectivity between the right posterior insula and the postcentral gyrus, compared to controls. As mentioned above, the posterior insula is functionally connected to these sensorimotor regions. Taken together, these results suggest that processing of somatosensory information could be impacted by traumatic experiences. Furthermore, sensorimotor circuits subserve the comprehension of phonological information, semantic categories, and grammar (Pulvermüller & Fadiga, 2010), suggesting that rsFC changes in these sensorimotor regions could have an impact on language skills.

The resting‐state networks defined by using the identified clusters as seeds highly overlapped with the somatomotor network. Behroozmand et al. (2015) identified a network involved in speech production and motor control in an fMRI study, which is concordant with the regions in Network 1.

### The interaction between sex and the number of TEs

We identified three clusters that showed significant sex differences in the association between the number of TEs and rsFC. These clusters were located within bilateral PCC and middle cingulate cortices and the right precuneus. PCC is a central region in the DMN and involved in autobiographical and episodic memory retrieval (Maddock et al., 2001; Natu et al., 2019). Altered rsFC between the PCC and other brain regions has been reported in both healthy adults with traumatic exposure (Philip, Sweet, et al., 2013) and patients with PTSD (Bluhm et al., 2009). For healthy adults with early life stress, decreased rsFC has been observed between the PCC and the medial prefrontal cortex, but sex differences were not examined in this study (Philip, Sweet, et al., 2013). Bluhm et al. (2009) observed lower positive functional connectivity between posterior cingulate cortex and the precuneus/bilateral lateral parietal cortices among 17 female patients with PTSD compared to 15 healthy controls, which is in concordance with our finding that females showed decreased rsFC within the identified clusters as the number of TEs increased. Functional connectivity between PCC and salience network regions (insula, anterior cingulate cortex) has also been associated with episodic memory performance (Viard et al., 2019). Intrusive memories such as flashbacks and nightmares are symptoms of PTSD, and sex differences in intrusive memories following trauma have been observed. Both females with PTSD and trauma‐exposed females displayed greater recall and reported more negative intrusive memories than males (Hsu et al., 2018).

The clusters with sex differences were mainly located within the DMN. In addition, the resting‐state networks defined by using these clusters as seeds also highly overlapped with the DMN. The DMN is a large intrinsic functional network. Its functions are associated with self‐oriented processing (Gusnard et al., 2001) and preparing for responses to environmental stimuli (Raichle & Gusnard, 2005). Numerous studies have reported decreased rsFC in the DMN among non‐psychiatric individuals with trauma exposure (Lu et al., 2017; Philip, Sweet, et al., 2013) and in patients with PTSD relative to controls (Bluhm et al., 2009; Tursich et al., 2015). However, sex‐specific differences in rsFC have not been well‐documented. Our analyses used a large youth population dataset with 57.8% females, providing power to identify significant sex‐specific associations. We observed that within these identified clusters, females showed decreased functional connectivity, whereas males showed increased connectivity as the number of TEs increased. This is consistent with previous findings of decreased rsFC within the DMN in patients with PTSD (Bluhm et al., 2009; Tursich et al., 2015), and the heightened risk for PTSD in females after experiencing trauma (Tolin & Foa, 2008).

The findings of this study reinforce previously identified brain linkages between experiencing traumatic events in childhood and long‐standing effects on language (Sylvestre et al., 2016) and somatosensory processing (Shepherd & Wild, 2014), as well as differences in intrusive memory recall (Hsu et al., 2018) and risk of PTSD between the sexes (Tolin & Foa, 2008). Future research, perhaps as part of longitudinal studies of children and adolescents currently underway or in development, could begin to establish causality.

### Limitations

There are several limitations associated with the current study. First, the average scan duration of resting‐state fMRI after quality control was less than 6 min. The limited scan duration may affect the reliability of functional connectivity (Noble et al., 2019). Second, the traumatic experience assessment was based on questions in a self‐reported PTSD scale. Self‐reported traumatic experiences might not reflect actual traumatic exposure. In addition, the PTSD scale was not a standard scale to measure childhood trauma, such as the Childhood Trauma Questionnaire (Bernstein et al., 2003). More stringent measurements are needed in future studies. Third, information regarding traumatic experiences is limited. We only examined the number of TEs, but did not evaluate trauma type, timing, duration, or severity. Sex‐specific associations observed in this study may have arisen due to potentially different trauma types experienced by males and females. Further studies are needed to verify our results and disentangle the association between these specific aspects of traumatic experiences and rsFC. Fourth, the ICD parameters captured a whole‐brain assessment of the connectivity between each voxel and all other voxels within the gray matter mask. Therefore, we cannot conclude that rsFC alterations were restricted to only the somatomotor network or the DMN, as voxels outside of these networks may have contributed to the observed changes in rsFC. This limitation could be addressed in future studies that focus specifically on connectivity within each of these networks. Fifth, although the PNC participants were not seeking help for psychiatric issues, some of the participants in our study might have developed PTSD or other mental disorders. These participants may have distorted our results, as we aimed to test our hypotheses in a non‐psychiatric population. Finally, we cannot infer causality based on this cross‐sectional dataset, so it is difficult to attribute differences in rsFC specifically to traumatic experiences. Longitudinal data are needed to analyze the trajectories of change in rsFC following traumatic experiences.

## CONCLUSIONS

In a large sample of non‐psychiatric youth, the current study reports a significant association between traumatic experiences and reduced rsFC in brain regions corresponding with the somatomotor network. This study is also the first to identify sex‐specific associations between traumatic experiences and rsFC in brain regions corresponding with the DMN. Further research is needed to establish the causality of these associations and to evaluate how these associations are qualified by trauma type, timing, duration, and severity. The neural basis for linkages between experiencing traumatic events in childhood and long‐standing effects on language and somatosensory processing, as well as differences in intrusive memory recall and risk of PTSD between the sexes also requires further research.

## CONFLICT OF INTEREST

The authors have declared that they have no competing or potential conflicts of interest.

## ETHICS STATEMENT

Appropriate informed consent was obtained from all study participants. The University of Pennsylvania and the Children’s Hospital of Philadelphia approved all study procedures (Satterthwaite et al., 2016).

## AUTHOR CONTRIBUTIONS

Jeffrey R. Gruen, Jeffrey G. Malins, and Shiying Wang designed the study. Shiying Wang performed the analyses and drafted the manuscript. Jeffrey R. Gruen supervised the findings of this work. All authors discussed the results and contributed to the writing of the final manuscript.

## Supporting information

Supporting Information S1

## Data Availability

The data that support the findings of this study are openly available in dbGaP at https://www.ncbi.nlm.nih.gov/projects/gap/cgi‐bin/study.cgi?study_id=phs000607.v2.p2 through dbGaP accession phs000607.v2.p2.

## References

[jcv212049-bib-0001] Alarcón, G. , Cservenka, A. , Rudolph, M. D. , Fair, D. A. , & Nagel, B. J. (2015). Developmental sex differences in resting state functional connectivity of amygdala sub‐regions. NeuroImage, 115, 235–244. 25887261 10.1016/j.neuroimage.2015.04.013 PMC4461484

[jcv212049-bib-0002] American Psychiatric Association . (2013). Diagnostic and statistical manual of mental disorders (DSM‐5®). American Psychiatric Publishing.

[jcv212049-bib-0003] Barzilay, R. , Calkins, M. E. , Moore, T. M. , Wolf, D. H. , Satterthwaite, T. D. , Scott, J. C. , Benton, T. D. , Gur, R. C. , & Gur, R. E. (2019). Association between traumatic stress load, psychopathology, and cognition in the Philadelphia Neurodevelopmental Cohort. Psychological Medicine, 49(2), 325–334. 29655375 10.1017/S0033291718000880

[jcv212049-bib-0004] Behroozmand, R. , Shebek, R. , Hansen, D. R. , Oya, H. , Robin, D. A. , Howard, M. A., III , & Greenlee, J. D. (2015). Sensory–motor networks involved in speech production and motor control: An fMRI study. NeuroImage, 109, 418–428. 25623499 10.1016/j.neuroimage.2015.01.040 PMC4339397

[jcv212049-bib-0005] Bernstein, D. P. , Stein, J. A. , Newcomb, M. D. , Walker, E. , Pogge, D. , Ahluvalia, T. , Handelsman, L. , Medrano, M. , Desmond, D. , & Zule, W. (2003). Development and validation of a brief screening version of the Childhood Trauma Questionnaire. Child Abuse & Neglect, 27(2), 169–190. 12615092 10.1016/s0145-2134(02)00541-0

[jcv212049-bib-0006] Bluhm, R. L. , Williamson, P. C. , Osuch, E. A. , Frewen, P. A. , Stevens, T. K. , Boksman, K. , Neufeld, R. W. , Théberge, J. , & Lanius, R. A. (2009). Alterations in default network connectivity in posttraumatic stress disorder related to early‐life trauma. Journal of Psychiatry & Neuroscience: JPN, 34(3), 187. 19448848 PMC2674971

[jcv212049-bib-0007] Briere, J. , & Elliott, D. M. (2003). Prevalence and psychological sequelae of self‐reported childhood physical and sexual abuse in a general population sample of men and women. Child Abuse & Neglect, 27(10), 1205–1222. 14602100 10.1016/j.chiabu.2003.09.008

[jcv212049-bib-0008] Calkins, M. E. , Moore, T. M. , Merikangas, K. R. , Burstein, M. , Satterthwaite, T. D. , Bilker, W. B. , Chiavacci, R. , Wolf, D. H. , Mentch, F. , Qiu, H. , Connolly, J. J. , Sleiman, P. A. , Hakonarson, H. , Gur, R. C. , & Gur, R. E. (2014). The psychosis spectrum in a young US community sample: Findings from the Philadelphia Neurodevelopmental Cohort. World Psychiatry, 13(3), 296–305. 25273303 10.1002/wps.20152 PMC4219071

[jcv212049-bib-0009] Copeland, W. E. , Keeler, G. , Angold, A. , & Costello, E. J. (2007). Traumatic events and posttraumatic stress in childhood. Archives of General Psychiatry, 64(5), 577–584. 17485609 10.1001/archpsyc.64.5.577

[jcv212049-bib-0010] Cox, R. W. (1996). Afni: Software for analysis and visualization of functional magnetic resonance neuroimages. Computers and Biomedical Research, 29(3), 162–173. 8812068 10.1006/cbmr.1996.0014

[jcv212049-bib-0011] Deen, B. , Pitskel, N. B. , & Pelphrey, K. A. (2011). Three systems of insular functional connectivity identified with cluster analysis. Cerebral Cortex, 21(7), 1498–1506. 21097516 10.1093/cercor/bhq186 PMC3116731

[jcv212049-bib-0012] Felmingham, K. , Williams, L. M. , Kemp, A. H. , Liddell, B. , Falconer, E. , Peduto, A. , & Bryant, R. (2010). Neural responses to masked fear faces: Sex differences and trauma exposure in posttraumatic stress disorder. Journal of Abnormal Psychology, 119(1), 241–247. 20141261 10.1037/a0017551

[jcv212049-bib-0013] Gusnard, D. A. , Akbudak, E. , Shulman, G. L. , & Raichle, M. E. (2001). Medial prefrontal cortex and self‐referential mental activity: Relation to a default mode of brain function. Proceedings of the National Academy of Sciences of the United States of America, 98(7), 4259–4264. 11259662 10.1073/pnas.071043098 PMC31213

[jcv212049-bib-0014] Hakonarson, H. , & Gur, R. E. (2017). Philadelphia Neurodevelopmental Cohort (Version 2); phs000607.v2.p2. dbGap.

[jcv212049-bib-0015] Hsu, C.‐M. K. , Kleim, B. , Nicholson, E. L. , Zuj, D. V. , Cushing, P. J. , Gray, K. E. , Clark, L. , & Felmingham, K. L. (2018). Sex differences in intrusive memories following trauma. PLoS One, 13(12), e0208575. 30521618 10.1371/journal.pone.0208575 PMC6283557

[jcv212049-bib-0016] Huang, A. W. , & Barber, A. D. (2021). Development of lateral pulvinar resting state functional connectivity and its role in attention. Cortex, 136, 77–88. 33486158 10.1016/j.cortex.2020.12.004 PMC8204675

[jcv212049-bib-0017] Jalbrzikowski, M. , Liu, F. , Foran, W. , Roeder, K. , Devlin, B. , & Luna, B. (2020). Resting‐state functional network organization is stable across adolescent development for typical and psychosis spectrum youth. Schizophrenia Bulletin, 46(2), 395–407. 31424081 10.1093/schbul/sbz053 PMC7442350

[jcv212049-bib-0018] Kilpatrick, D. G. , Ruggiero, K. J. , Acierno, R. , Saunders, B. E. , Resnick, H. S. , & Best, C. L. (2003). Violence and risk of PTSD, major depression, substance abuse/dependence, and comorbidity: Results from the National Survey of Adolescents. Journal of Consulting and Clinical Psychology, 71(4), 692–700. 12924674 10.1037/0022-006x.71.4.692

[jcv212049-bib-0019] Lu, S. , Gao, W. , Wei, Z. , Wang, D. , Hu, S. , Huang, M. , Xu, Y. , & Li, L. (2017). Intrinsic brain abnormalities in young healthy adults with childhood trauma: A resting‐state functional magnetic resonance imaging study of regional homogeneity and functional connectivity. Australian and New Zealand Journal of Psychiatry, 51(6), 614–623. 27694638 10.1177/0004867416671415

[jcv212049-bib-0020] Maddock, R. J. , Garrett, A. S. , & Buonocore, M. H. (2001). Remembering familiar people: The posterior cingulate cortex and autobiographical memory retrieval. Neuroscience, 104(3), 667–676. 11440800 10.1016/s0306-4522(01)00108-7

[jcv212049-bib-0021] Majer, M. , Nater, U. M. , Lin, J.‐M. S. , Capuron, L. , & Reeves, W. C. (2010). Association of childhood trauma with cognitive function in healthy adults: A pilot study. BMC Neurology, 10(1), 61. 20630071 10.1186/1471-2377-10-61 PMC2910667

[jcv212049-bib-0022] Marusak, H. A. , Etkin, A. , & Thomason, M. E. (2015). Disrupted insula‐based neural circuit organization and conflict interference in trauma‐exposed youth. NeuroImage, 8, 516–525. 26199869 10.1016/j.nicl.2015.04.007 PMC4477108

[jcv212049-bib-0023] Natu, V. S. , Lin, J.‐J. , Burks, A. , Arora, A. , Rugg, M. D. , & Lega, B. (2019). Stimulation of the posterior cingulate cortex impairs episodic memory encoding. Journal of Neuroscience, 39(36), 7173–7182. 31358651 10.1523/JNEUROSCI.0698-19.2019 PMC6733540

[jcv212049-bib-0024] Noble, S. , Scheinost, D. , & Constable, R. T. (2019). A decade of test‐retest reliability of functional connectivity: A systematic review and meta‐analysis. NeuroImage, 203, 116157. 31494250 10.1016/j.neuroimage.2019.116157 PMC6907736

[jcv212049-bib-0025] Pérez‐Fuentes, G. , Olfson, M. , Villegas, L. , Morcillo, C. , Wang, S. , & Blanco, C. (2013). Prevalence and correlates of child sexual abuse: A national study. Comprehensive Psychiatry, 54(1), 16–27. 22854279 10.1016/j.comppsych.2012.05.010 PMC3518746

[jcv212049-bib-0026] Philip, N. S. , Kuras, Y. I. , Valentine, T. R. , Sweet, L. H. , Tyrka, A. R. , Price, L. H. , & Carpenter, L. L. (2013). Regional homogeneity and resting state functional connectivity: Associations with exposure to early life stress. Psychiatry Research: Neuroimaging, 214(3), 247–253. 10.1016/j.pscychresns.2013.07.013 PMC3849340 24090510

[jcv212049-bib-0027] Philip, N. S. , Sweet, L. H. , Tyrka, A. R. , Price, L. H. , Bloom, R. F. , & Carpenter, L. L. (2013). Decreased default network connectivity is associated with early life stress in medication‐free healthy adults. European Neuropsychopharmacology, 23(1), 24–32. 23141153 10.1016/j.euroneuro.2012.10.008 PMC3581700

[jcv212049-bib-0028] Pulvermüller, F. , & Fadiga, L. (2010). Active perception: Sensorimotor circuits as a cortical basis for language. Nature Reviews Neuroscience, 11(5), 351–360. 20383203 10.1038/nrn2811

[jcv212049-bib-0029] Raichle, M. E. , & Gusnard, D. A. (2005). Intrinsic brain activity sets the stage for expression of motivated behavior. Journal of Comparative Neurology, 493(1), 167–176. 16254998 10.1002/cne.20752

[jcv212049-bib-0030] Saad, Z. S. , Reynolds, R. C. , Argall, B. , Japee, S. , & Cox, R. W. (2004). Suma: An interface for surface‐based intra‐and inter‐subject analysis with AFNI. Paper presented at the 2004 2nd IEEE International Symposium on Biomedical Imaging: Nano to Macro (IEEE Cat No. 04EX821), Arlington, VA.

[jcv212049-bib-0031] Satterthwaite, T. D. , Connolly, J. J. , Ruparel, K. , Calkins, M. E. , Jackson, C. , Elliott, M. A. , Hopson, R. , Prabhakaran, K. , Behr, M. , Qiu, H. , Mentch, F. D. , Chiavacci, R. , Sleiman, P. M. A. , Gur, R. C. , Hakonarson, H. , & Gur, R. E. (2016). The Philadelphia Neurodevelopmental Cohort: A publicly available resource for the study of normal and abnormal brain development in youth. NeuroImage, 124, 1115–1119. 25840117 10.1016/j.neuroimage.2015.03.056 PMC4591095

[jcv212049-bib-0032] Satterthwaite, T. D. , Wolf, D. H. , Roalf, D. R. , Ruparel, K. , Erus, G. , Vandekar, S. , Elliott, M. A. , Smith, A. , Hakonarson, H. , Verma, R. , Davatzikos, C. , Gur, R. E. , & Gur, R. C. (2015). Linked sex differences in cognition and functional connectivity in youth. Cerebral Cortex, 25(9), 2383–2394. 24646613 10.1093/cercor/bhu036 PMC4537416

[jcv212049-bib-0033] Scheinost, D. , Benjamin, J. , Lacadie, C. , Vohr, B. , Schneider, K. C. , Ment, L. R. , Papademetris, X. , & Constable, R. T. (2012). The intrinsic connectivity distribution: A novel contrast measure reflecting voxel level functional connectivity. NeuroImage, 62(3), 1510–1519. 22659477 10.1016/j.neuroimage.2012.05.073 PMC3538880

[jcv212049-bib-0034] Shepherd, L. , & Wild, J. (2014). Emotion regulation, physiological arousal and PTSD symptoms in trauma‐exposed individuals. Journal of Behavior Therapy and Experimental Psychiatry, 45(3), 360–367. 24727342 10.1016/j.jbtep.2014.03.002 PMC4053589

[jcv212049-bib-0035] Sheynin, J. , Duval, E. R. , Lokshina, Y. , Scott, J. C. , Angstadt, M. , Kessler, D. , Gur, R. E. , Gur, R. C. , & Liberzon, I. (2020). Altered resting‐state functional connectivity in adolescents is associated with PTSD symptoms and trauma exposure. NeuroImage, 26, 102215. 32339825 10.1016/j.nicl.2020.102215 PMC7184176

[jcv212049-bib-0036] Sripada, R. K. , King, A. P. , Welsh, R. C. , Garfinkel, S. N. , Wang, X. , Sripada, C. S. , & Liberzon, I. (2012). Neural dysregulation in posttraumatic stress disorder: Evidence for disrupted equilibrium between salience and default mode brain networks. Psychosomatic Medicine, 74(9), 904–911. 23115342 10.1097/PSY.0b013e318273bf33 PMC3498527

[jcv212049-bib-0037] Sylvestre, A. , Bussières, È.‐L. , & Bouchard, C. (2016). Language problems among abused and neglected children: A meta‐analytic review. Child Maltreatment, 21(1), 47–58. 26620719 10.1177/1077559515616703

[jcv212049-bib-0038] Thomas Yeo, B. , Krienen, F. M. , Sepulcre, J. , Sabuncu, M. R. , Lashkari, D. , Hollinshead, M. , Smoller, J. W. , Zöllei, L. , Polimeni, J. R. , Fischl, B. , Liu, H. , & Buckner, R. L. (2011). The organization of the human cerebral cortex estimated by intrinsic functional connectivity. Journal of Neurophysiology, 106(3), 1125–1165. 21653723 10.1152/jn.00338.2011 PMC3174820

[jcv212049-bib-0039] Tolin, D. F. , & Foa, E. B. (2008). Sex differences in trauma and posttraumatic stress disorder: A quantitative review of 25 years of research. Psychological Bulletin, 132, 959–992. 10.1037/0033-2909.132.6.959 17073529

[jcv212049-bib-0040] Tursich, M. , Ros, T. , Frewen, P. , Kluetsch, R. , Calhoun, V. D. , & Lanius, R. A. (2015). Distinct intrinsic network connectivity patterns of post‐traumatic stress disorder symptom clusters. Acta Psychiatrica Scandinavica, 132(1), 29–38. 25572430 10.1111/acps.12387

[jcv212049-bib-0041] Uddin, L. Q. , Nomi, J. S. , Hebert‐Seropian, B. , Ghaziri, J. , & Boucher, O. (2017). Structure and function of the human insula. Journal of Clinical Neurophysiology: Official Publication of the American Electroencephalographic Society, 34(4), 300–306. 28644199 10.1097/WNP.0000000000000377 PMC6032992

[jcv212049-bib-0042] Viard, A. , Mutlu, J. , Chanraud, S. , Guenolé, F. , Egler, P.‐J. , Gérardin, P. , Dayan, J. , Eustache, F. , & Guillery‐Girard, B. (2019). Altered default mode network connectivity in adolescents with post‐traumatic stress disorder. NeuroImage, 22, 101731. 30831461 10.1016/j.nicl.2019.101731 PMC6402428

[jcv212049-bib-0043] Wu, Y. , Li, H. , Zhou, Y. , Yu, J. , Zhang, Y. , Song, M. , Qin, W. , Jiang, T. (2016). Sex‐specific neural circuits of emotion regulation in the centromedial amygdala. Scientific Reports, 6(1), 1–10. 27004933 10.1038/srep23112 PMC4804331

[jcv212049-bib-0044] Zhang, Y. , Xie, B. , Chen, H. , Li, M. , Guo, X. , & Chen, H. (2016). Disrupted resting‐state insular subregions functional connectivity in post‐traumatic stress disorder. Medicine, 95(27), e4083. 27399097 10.1097/MD.0000000000004083 PMC5058826

