# Supplementary material for: Sex‐specific associations between traumatic experiences and resting‐state functional connectivity in the Philadelphia Neurodevelopmental Cohort
Source: JCPP Adv. 2021 Dec 6;1(4):e12049. doi: 10.1002/jcv2.12049 (PMC8713563; doi:10.1002/jcv2.12049)
Supplement: Supplementary file 1 — Supporting Information S1 [file JCV2-1-e12049-s001.docx]

**Supporting Information - Sex-Specific Associations Between Traumatic Experiences and Resting-State Functional Connectivity in the Philadelphia Neurodevelopmental Cohort**

**Appendix S1. Traumatic experience assessment**

Nine questions from the PTSD assessment scale (TableS1) in GOASSESS (Calkins et al., 2014) were used to assess traumatic experiences. Responses for each question were assigned a value of 1 for yes, and 0 for no. Questions 4 and 5 probed sexual assault. Most subjects left question 5 unanswered, which asked about rape, so we combined questions 4 and 5 to capture as much information as possible about sexual assault. We assigned a value of 1 when the answer to either question 4 or 5 was yes. Then, for each participant, we summed the number of unweighted traumatic events. Because the number of participants who experienced three or more TEs was relatively small, this could have resulted in unstable estimation of effect size in the regression analysis. Therefore, we grouped these participants into one group, and the number of TEs was coded as 0, 1, 2, or 3 for use in subsequent regression analyses.

**Appendix S2. Image acquisition**

MRI images were collected using a single 3T Siemens TIM Trio whole-body scanner located in the Hospital of the University of Pennsylvania (Satterthwaite et al., 2016). Anatomical images were acquired using a magnetization prepared, rapid-acquisition gradient-echo (MPRAGE) sequence (matrix size=256x192; voxel size=0.9x0.9x1 mm; field-of-view (FoV)=180x240 mm; repetition time (TR)=1810 ms; echo time (TE)=3.5 ms; flip angle=9$^{\circ}$). Resting-state BOLD scans were obtained using a single-shot, interleaved multi-slice, gradient-echo, echo planar imaging (GE-EPI) sequence (matrix size=64x64; voxel size=3x3x3 mm; FoV=192x192 mm; TR=3000 ms; TE=32 ms; flip angle=90$^{\circ}$). The total duration of the resting-state scan was 6.2 min. Participants were asked to keep their eyes open, stay awake, fixate on the displayed crosshair, and remain still.

**Appendix S3. Outlier voxels**

Outliers were defined as follows:

The trend of general linear model and median absolute deviation (MAD) was calculated, where MAD was defined as the median absolute value of time series minus trend. In the time series, points that are “far away” from the trend of the general linear model are called outliers, where “far” is defined by:

$\alpha*\sqrt{\frac{\pi}{2}}*MAD$,

where $\alpha=qginv\left( \frac{0.001}{N} \right)$ (inverse of reversed Gaussian cumulative density function), and $N$ is the length of the time series. In AFNI, *3dToutcount* was used to calculate the number of outlier voxels for each volume.

**Appendix S4. Tissue-based regression**

Tissue-based regression was performed to regress out the average signals of individual eroded white matter masks and the first three principal components of individual eroded lateral ventricle masks using the “fast” ANATICOR program in AFNI (Jo, Saad, Simmons, Milbury, & Cox, 2010). Freesurfer (Dale, Fischl, & Sereno, 1999) was used to generate white matter and lateral ventricle masks for each participant based on anatomical scans and using the Desikan-Killiany atlas (Desikan et al., 2006).

**Appendix S5. Intrinsic connectivity distribution (ICD)**

ICD is a method to measure voxel-level functional connectivity, as described by Scheinost et al. (Scheinost et al., 2012). Correlation coefficients were calculated between each voxel and every other voxel in the brain using time course residuals from the general linear model. Then, positive correlation coefficients were used to generate a histogram. The survival function computed from this histogram was approximated using a Weibull distribution model $S\left( r \right)=e^{-\alpha*r^{\beta}}$. The survival function was characterized by a variance parameter alpha and a shape parameter beta. A smaller alpha and a larger beta for any voxel represent a relatively larger amount of strong connections and higher connectivity with other voxels.

**Table S1. Questions to assess traumatic experiences**

|  | **Questions** | |
| --- | --- | --- |
| 1 | Have you ever been in a flood or a tornado or an earthquake or a hurricane or some other natural disaster where you thought you were going to die or be seriously hurt? | |
| 2 | Have you ever been in a situation where you thought you or someone close to you was going to be killed or be hurt very badly? |  |
| 3 | Have you ever been attacked by somebody or badly beaten? |  |
| 4 | Have you ever been very upset by someone forcing you to do something sexual? |  |
| 5 | Have you ever been attacked sexually or raped? |  |
| 6 | Have you ever been threatened with a weapon? |  |
| 7 | Have you ever been in a bad accident? |  |
| 8 | Other than television or at the movies, have you ever seen or heard somebody get killed or get hurt very badly or die? |  |
| 9 | Have you ever been very upset by seeing a dead body or by seeing pictures of the dead body of somebody you knew well? |  |

**Table S2. Demographic information and the number of TEs for included and excluded participants**

| **Characteristics** | **Included (n=914)** | **Excluded** **(n=481)** | **Statistics (ANOVA** $\boldsymbol{F}$**/ Pearson** $\mathbf{X}^{\mathbf{2}}$**)** | ***P* value** |
| --- | --- | --- | --- | --- |
| Female sex, No. (%) | 529 (57.9) | 216 (44.9) | $X^{2}$=20.79 | <0.001 |
| Race |  |  | $X^{2}$=17.95 | <0.001 |
| African-American, No. (%) | 423 (46.3) | 166 (34.5) |  |  |
| European-American, No. (%) | 396 (43.3) | 252 (52.4) |  |  |
| Other, No. (%) | 91 (10.0) | 63 (13.1) |  |  |
| Age, mean (SD), years | 14.6 (3.32) | 14.1 (3.66) | *F*=5.75 | 0.017 |
| ME, mean (SD), years | 14.2 (2.43) | 14.5 (2.48) | *F*=6.15 | 0.013 |
| The number of TEs |  |  | $X^{2}$=0.174 | 0.982 |
| TE=0, No. (%) | 477 (52.2) | 244 (59.3) |  |  |
| TE=1, No. (%) | 219 (24.0) | 114 (27.3) |  |  |
| TE=2, No. (%) | 113 (12.4) | 63 (15.1) |  |  |
| TE$\geq$3, No. (%) | 105 (11.5) | 56 (13.4) |  |  |

Abbreviation: ME, years of maternal education; TE, traumatic event

**Table S3. Demographic information for participants by number of traumatic events**

|  | **Participants (n=914)** | | | |
| --- | --- | --- | --- | --- |
| **Characteristics** | **TE=0 (n=477)** | **TE=1 (n=219)** | **TE=2 (n=113)** | **TE**$\boldsymbol{\geq}$**3 (n=105)** |
| Female sex, No. (%) | 274 (57.4) | 133 (60.7) | 68 (60.1) | 54 (51.4) |
| Race |  |  |  |  |
| African-American, No. (%) | 187 (39.2) | 99 (45.2) | 66 (58.4) | 71 (67.6) |
| European-American, No. (%) | 233 (48.8) | 97 (44.3) | 45 (39.8) | 21 (20.0) |
| Other, No. (%) | 54 (11.3) | 22 (10.0) | 2 (1.8) | 13 (12.4) |
| Age, mean (SD), years | 13.9 (3.5) | 14.9 (3.0) | 15.2 (2.8) | 16.1 (2.9) |
| ME, mean (SD), years | 14.4 (2.4) | 14.0 (2.5) | 14.1 (2.6) | 13.4 (2.1) |

Abbreviations: TE, traumatic event; ME, years of maternal education

**Table S4. Demographic information for participants with and without traumatic exposure**

| **Characteristics** | **Non-exposure** **(n=477)** | **Exposure** **(n=437)** | **Statistics (ANOVA** $\boldsymbol{F}$**/ Pearson** $\mathbf{X}^{\mathbf{2}}$**)** | ***P* value** |
| --- | --- | --- | --- | --- |
| Female sex, No. (%) | 274 (57.4) | 255 (58.4) | $X^{2}$=0.045 | 0.83 |
| Race |  |  | $X^{2}$=19.7 | <0.001 |
| African-American, No. (%) | 187 (39.2) | 236 (54.0) |  |  |
| European-American, No. (%) | 233 (48.8) | 163 (37.3) |  |  |
| Other, No. (%) | 54 (11.3) | 37 (8.5) |  |  |
| Age, mean (SD), years | 13.9 (3.50) | 15.3 (2.94) | *F*=39.7 | <0.001 |
| ME, mean (SD), years | 14.4 (2.41) | 13.9 (2.42) | *F*=10.4 | 0.001 |

Abbreviation: ME, years of maternal education

**Table S5. Exposure of participants to each category of traumatic event**

| **Traumatic event** | **N (% of cohort)** | **Number of females in category (%)** |
| --- | --- | --- |
| 1. Experienced a natural disaster | 31 (3.4) | 20 (64.5) |
| 2. Thought you or someone close to you was going to be killed or be hurt very badly | 120 (13.1) | 66 (55.0) |
| 3. Attacked by somebody or badly beaten | 55 (6.0) | 26 (47.3) |
| 4. Sexually forced | 26 (2.8) | 22 (84.6) |
| 5. Threatened with a weapon | 60 (6.6) | 17 (28.4) |
| 6. Experienced a bad accident | 91 (10.0) | 52 (57.1) |
| 7. Witnessed someone getting killed, badly beaten, or die | 211 (23.1) | 124 (58.8) |
| 8. Upset by seeing a dead body or pictures of the dead body of somebody you knew well | 228 (24.9) | 137 (60.1) |

**Table S6. Summary of significant clusters that passed cluster correction in regression on ICD alpha values without adding interaction between sex and the number of TEs**

| **Cluster^a^** | **Size (voxels)** | **Hemisphere** | **Peak voxel^b^** | **Average effect size^c^** | **Regions (% of each region in cluster)** |
| --- | --- | --- | --- | --- | --- |
| **Significant clusters for the main effect of the number of TEs on ICD alpha values** | | | | | |
| 1 | 75 | Left | (-52.5, -22.5, 7.5) | 0.054 | superior temporal gyrus (61.5), Heschl’s gyrus (22.0), Rolandic operculum (16.2) |
| 2 | 50 | Right | (37.5, -25.5, 13.5) | 0.053 | Heschl’s gyrus (44.0), insula (28.2), superior temporal gyrus (8.8) |
| 3 | 32 | Left | (-4.5, -13.5, 55.5) | 0.057 | paracentral lobule (65.4), SMA (34.0) |
| 4 | 19 | Left | (-31.5, -28.5, 55.5) | 0.045 | precentral gyrus (61.6), postcentral gyrus (38.4) |

Abbreviation: SMA, supplementary motor area; TE, traumatic event.

^a^ Clusters 1 and 2 have cluster corrected *p* values << 0.01, Cluster 3 has a cluster corrected *p* value < 0.01, and Cluster 4 has a cluster corrected *p* value < 0.03.

^b^ Peak voxel: The coordinate of the voxel with the largest absolute regression coefficient value within each cluster in MNI152 space in LPI orientation.

^c^Average effect size: the average effect size of voxels within the cluster. For the main effect of the number of TEs, the effect size represents the change in alpha value as the number of TEs increases by one.

**Table S7. Summary of significant clusters that passed cluster correction in regression on ICD alpha values without adding covariates for age and maternal education**

| **Cluster^a^** | **Size (voxels)** | **Hemisphere** | **Peak voxel^b^** | **Average effect size^c^** | **Regions** **(% of each region in cluster)** |
| --- | --- | --- | --- | --- | --- |
| **Significant clusters for the main effect of the number of TEs on ICD alpha values** | | | | | |
| 1 | 152 | Left | (-52.5, -22.5, 7.5) | 0.065 | superior temporal gyrus (66.2), Heschl’s gyrus (9.9), Rolandic operculum (9.3), insula (7.5) |
| 2 | 51 | Right | (37.5, -25.5, 13.5) | 0.062 | Heschl’s gyrus (45.5), superior temporal gyrus (27.2), insula (15.3) |
| 3 | 22 | Left | (-31.5, -28.5, 55.5) | 0.052 | precentral gyrus (58.0), postcentral gyrus (42.0) |
| **Significant clusters for the interaction between sex and the number of TEs on ICD alpha values** | | | | | |
| 4 | 35 | Right | (7.5, -46.5, 13.5) | -0.12 | posterior cingulate cortex (42.2), middle cingulate cortex (31.9), precuneus (24.5), |
| 5 | 29 | Right | (7.5, -67.5, 34.5) | -0.13 | precuneus (83.4), cuneus (15.1) |
| 6 | 19 | Left | (-4.5, -37.5, 34.5) | -0.13 | posterior cingulate cortex (72.6), middle cingulate cortex (25.0) |

Abbreviation: SMA, supplementary motor area; TE, traumatic event.

^a^ Clusters 1 and 2 have cluster corrected *p* values << 0.01, Clusters 4 and 5 have cluster corrected *p* values < 0.01, Cluster 3 has a cluster corrected *p* value < 0.02, and Cluster 6 has a cluster corrected *p* value < 0.03.

^b^ Peak voxel: The coordinate of the voxel with the largest absolute regression coefficient value within each cluster in MNI152 space in LPI orientation.

^c^Average effect size: the average effect size of voxels within the cluster. For the main effect of the number of TEs, the effect size represents the change of alpha value as the number of TEs increases by one. For the interaction between sex and the number of TEs, the effect size represents the difference between males and females in the change of alpha values as the number of TEs increases by one.

**Table S8: Percentage of Network 1 and Network 5 that overlapped with the reference networks in Yeo et al. (2011).**

| **Network** | **Number of overlapping voxels in Network 1 (%)** | **Number of overlapping voxels in Network 5 (%)** |
| --- | --- | --- |
| Visual | 0 (0) | 259 (14.9) |
| Somatomotor | 1421 (82.5) | 8 (0.5) |
| Dorsal attention | 0 (0) | 51 (2.9) |
| Ventral attention | 563 (32.7) | 75 (4.3) |
| Limbic | 0 (0) | 2 (0.1) |
| Frontoparietal | 0 (0) | 455 (26.2) |
| Default mode | 1 (0.06) | 1391 (80.1) |

**Table S9. Brain regions in resting-state Network 2**

| **Hemisphere** | **Regions** |
| --- | --- |
| Bilateral | superior temporal gyrus, Heschl’s gyrus, SMA, middle cingulate cortex, precentral gyrus, postcentral gyrus, Rolandic operculum, insula |
| Right | paracentral lobule |

**Table S10. Brain regions in resting-state Network 3**

| **Hemisphere** | **Regions** |
| --- | --- |
| Bilateral | SMA, paracentral lobule, precentral gyrus, postcentral gyrus |

**Table S11. Brain regions in resting-state Network 4**

| **Hemisphere** | **Regions** |
| --- | --- |
| Bilateral | paracentral lobule, precentral gyrus, postcentral gyrus, SMA |

**Table S12. Brain regions in resting-state Network 6**

| **Hemisphere** | **Regions** |
| --- | --- |
| Bilateral | precuneus, middle cingulate cortex, cuneus, posterior cingulate cortex, angular gyrus |
| Left | middle occipital gyrus |
| Right | superior frontal gyrus, middle frontal gyrus, mid-orbital gyrus, anterior cingulate cortex |

**Table S13. Brain regions in resting-state Network 7**

| **Hemisphere** | **Regions** |
| --- | --- |
| Bilateral | precuneus, middle cingulate cortex, posterior cingulate cortex, mid-orbital gyrus, angular gyrus, anterior cingulate cortex |

**Fig S1. Conjunction map for significant clusters of age effect and the main effect of the number of TEs after cluster correction.**


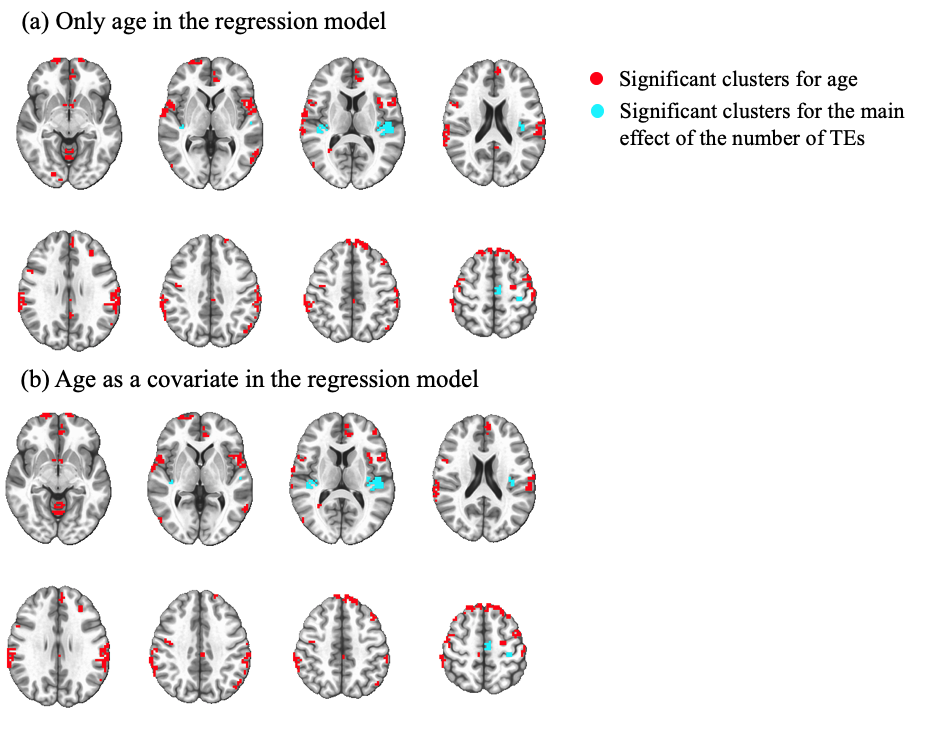


(a) Conjunction map of significant clusters for the age effect when ICD alpha values were regressed on age only (red) and the main effect of the number of TEs when ICD alpha values were regressed on age, maternal education, sex, the number of TEs, and the interaction between sex and the number of TEs (teal). (b) Conjunction map of significant clusters for the age effect (red) and the main effect of the number of TEs in model where ICD alpha values were regressed on age, maternal education, sex, the number of TEs, and the interaction between sex and the number of TEs (teal). Cluster correction was performed with the voxel-wise threshold set at *p* = 0.001, cluster corrected at 𝛼 = 0.05. The space between layers is 9 mm.

**Fig S2. Conjunction map for significant clusters of age effect and the interaction between the number of TEs and sex after cluster correction.**

**
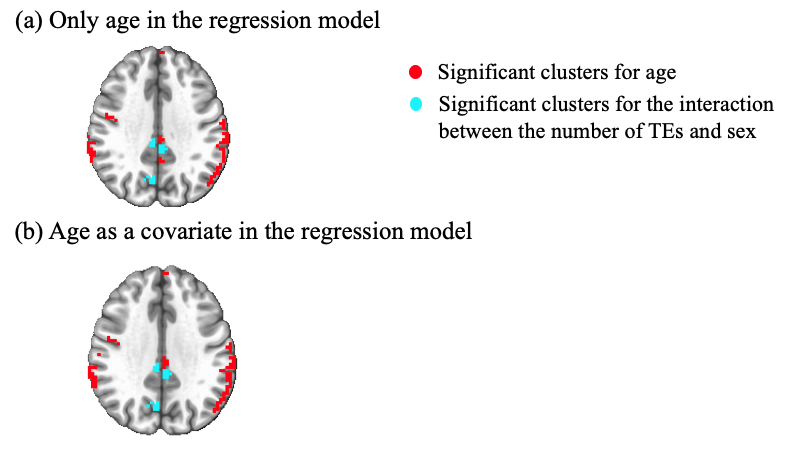
**

(a) Conjunction map of significant clusters for the age effect when ICD alpha values were regressed on age only (red) and the interaction between the number of TEs and sex when ICD alpha values were regressed on age, maternal education, sex, the number of TEs, and the interaction between sex and the number of TEs (teal). (b) Conjunction map of significant clusters for the age effect (red) and the interaction between the number of TEs and sex in model where ICD alpha values were regressed on age, maternal education, sex, the number of TEs, and the interaction between sex and the number of TEs (teal). Cluster correction was performed with the voxel-wise threshold set at *p* = 0.001, cluster corrected at 𝛼 = 0.05.

**Fig S3. Peak nodes within Network 1**


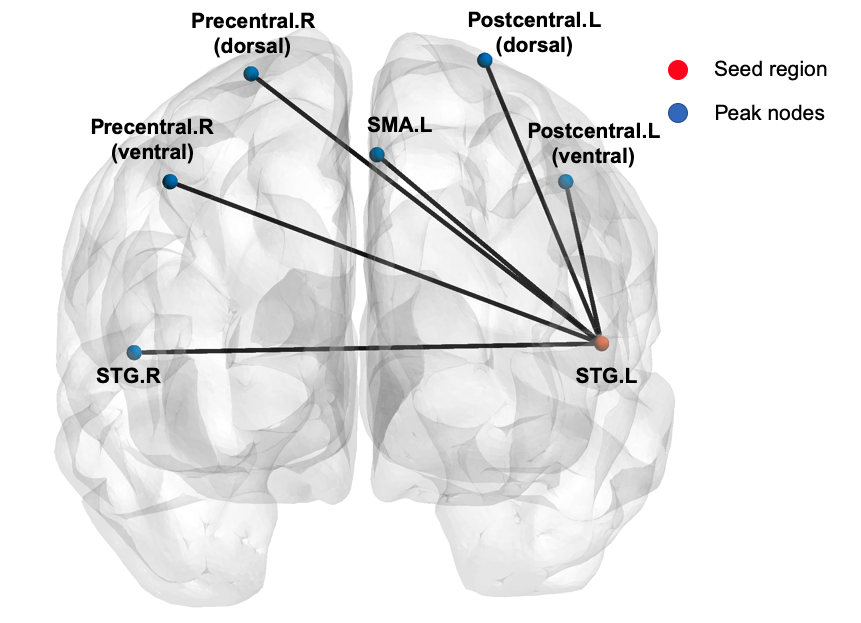


Peak nodes whose resting-state time courses were highly correlated with Cluster 1 within Network 1. Peak nodes were extracted using AFNI (Cox, 1996) (*3dExtrema*; minimum separation distance of 30 mm, or 10 voxels). BrainNet Viewer (Xia, Wang, & He, 2013) was used for visualization. STG: superior temporal gyrus, SMA: supplementary motor area, R: right hemisphere, L: left hemisphere.

**Fig S4. Resting-state Network 2**


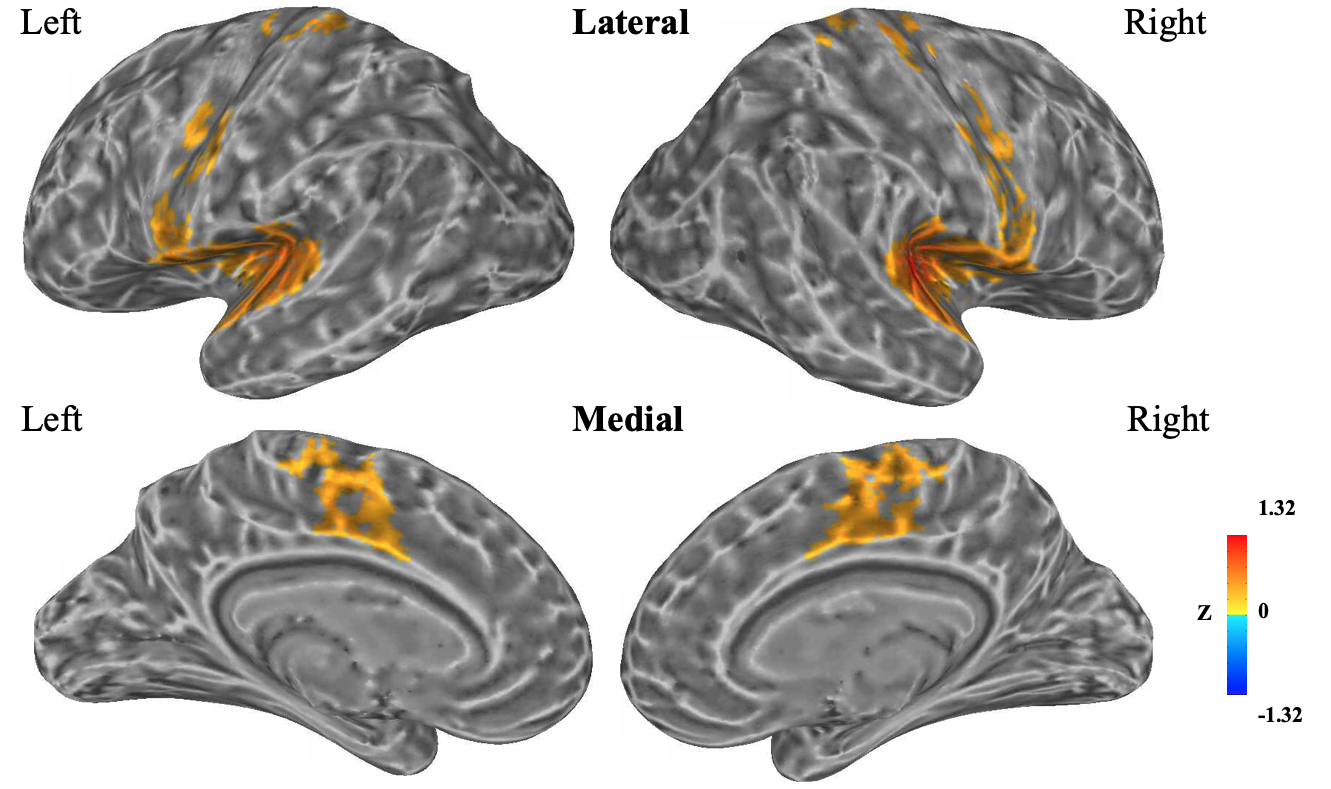


Resting-state Network 2 defined by using Cluster 2 as a seed region. The correlation coefficients between the average time-series data of the seed region with every other voxel were calculated and transformed to z scores. A *t*-test identified significant regions (*p* < 1x10^-44^, *FDR* < 3x10^-16^) that defined a resting-state network. Colors represent the value of z scores within the defined resting-state network.

**Fig S5. Resting-state Network 3**


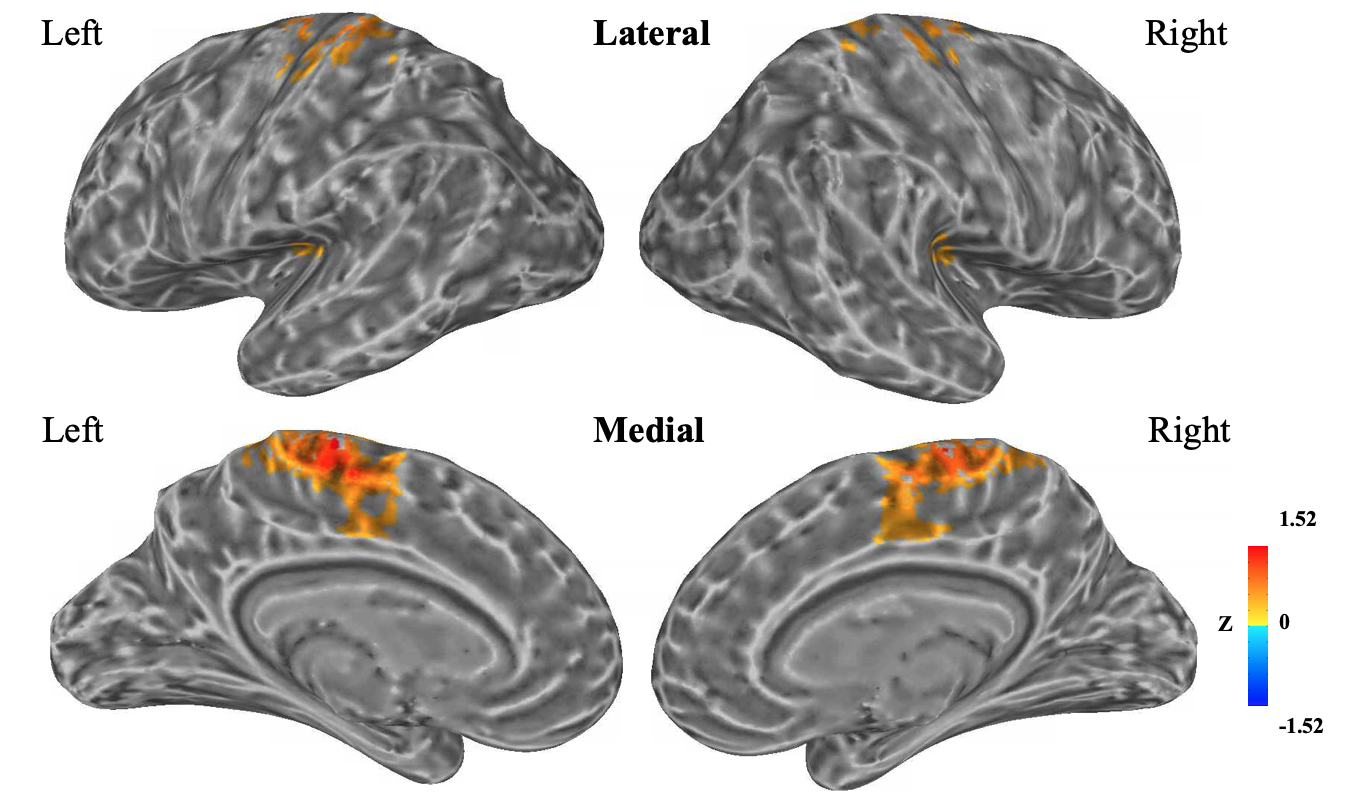


Resting-state Network 3 defined by using Cluster 3 as a seed region. The correlation coefficients between the average time-series data of the seed region with every other voxel were calculated and transformed to z scores. A *t*-test identified significant regions (*p* < 1x10^-44^, *FDR* < 3x10^-16^) that defined a resting-state network. Colors represent the value of z scores within the defined resting-state network.

**Fig S6. Resting-state Network 4**


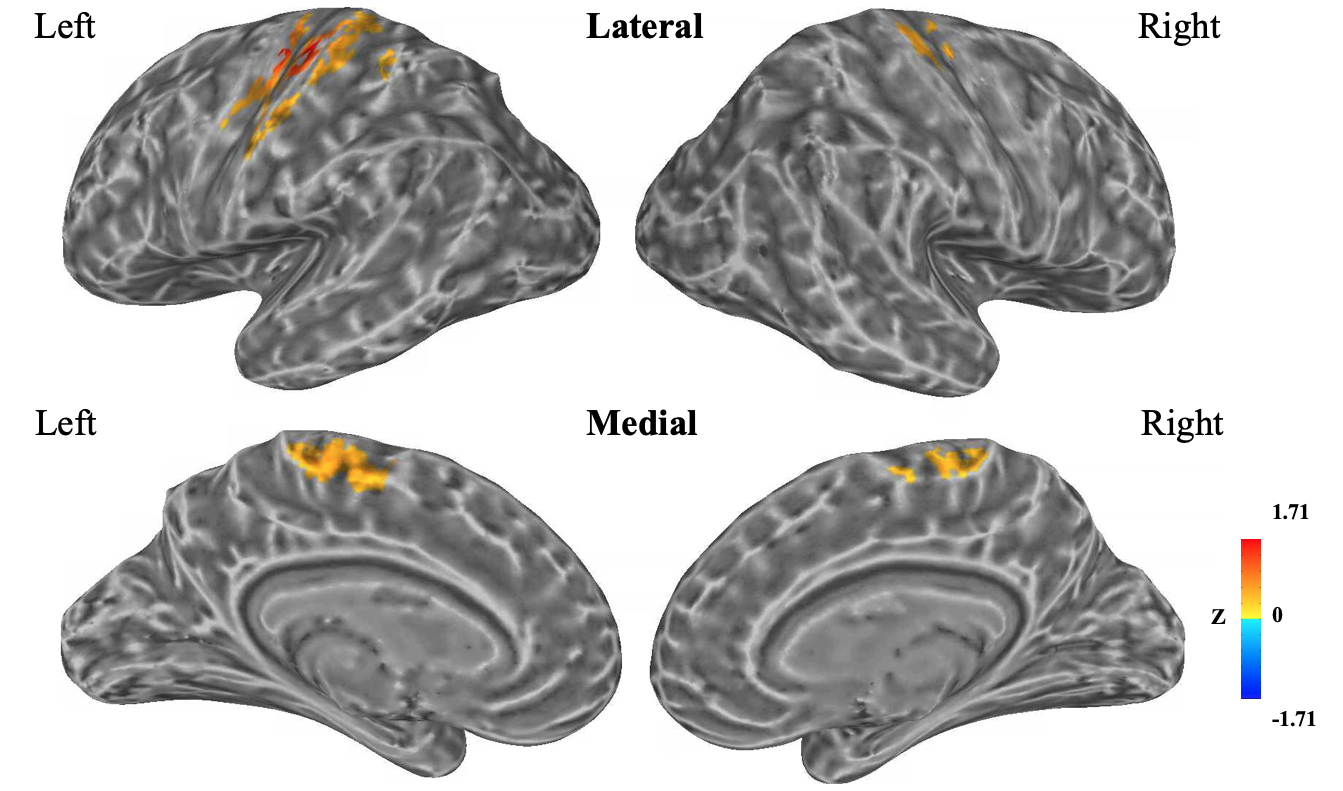


Resting-state Network 4 defined by using Cluster 4 as a seed region. The correlation coefficients between the average time-series data of the seed region with every other voxel were calculated and transformed to z scores. A *t*-test identified significant regions (*p* < 1x10^-44^, *FDR* < 3x10^-16^) that defined a resting-state network. Colors represent the value of z scores within the defined resting-state network.

**Fig S7. Peak nodes within Network 5**


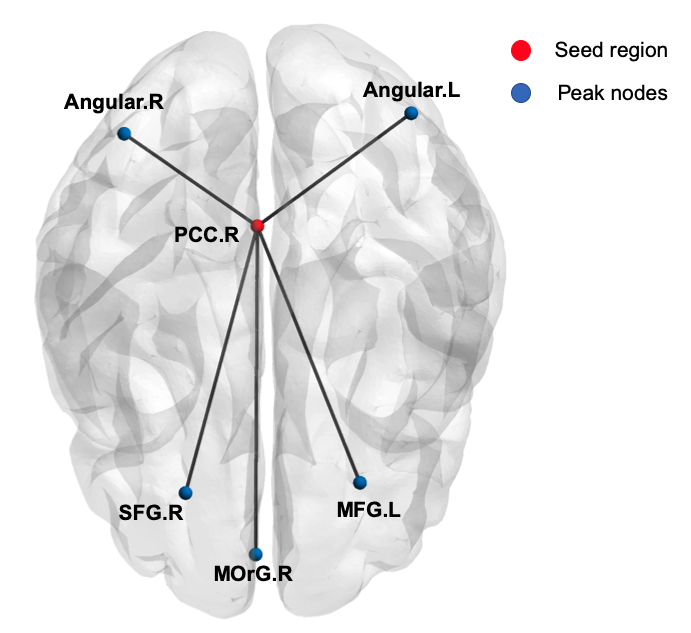


Peak nodes whose resting-state time courses were highly correlated with Cluster 5 within Network 5. Peak nodes were extracted using AFNI (Cox, 1996) (*3dExtrema*; minimum separation distance of 30 mm, or 10 voxels). BrainNet Viewer (Xia et al., 2013) was used for visualization. PCC: posterior cingulate cortex, MFG: middle frontal gyrus, SFG: superior frontal gyrus, MOrG: medial-orbital gyrus, R: right hemisphere, L: left hemisphere.

**Fig S8. Resting-state Network 6**


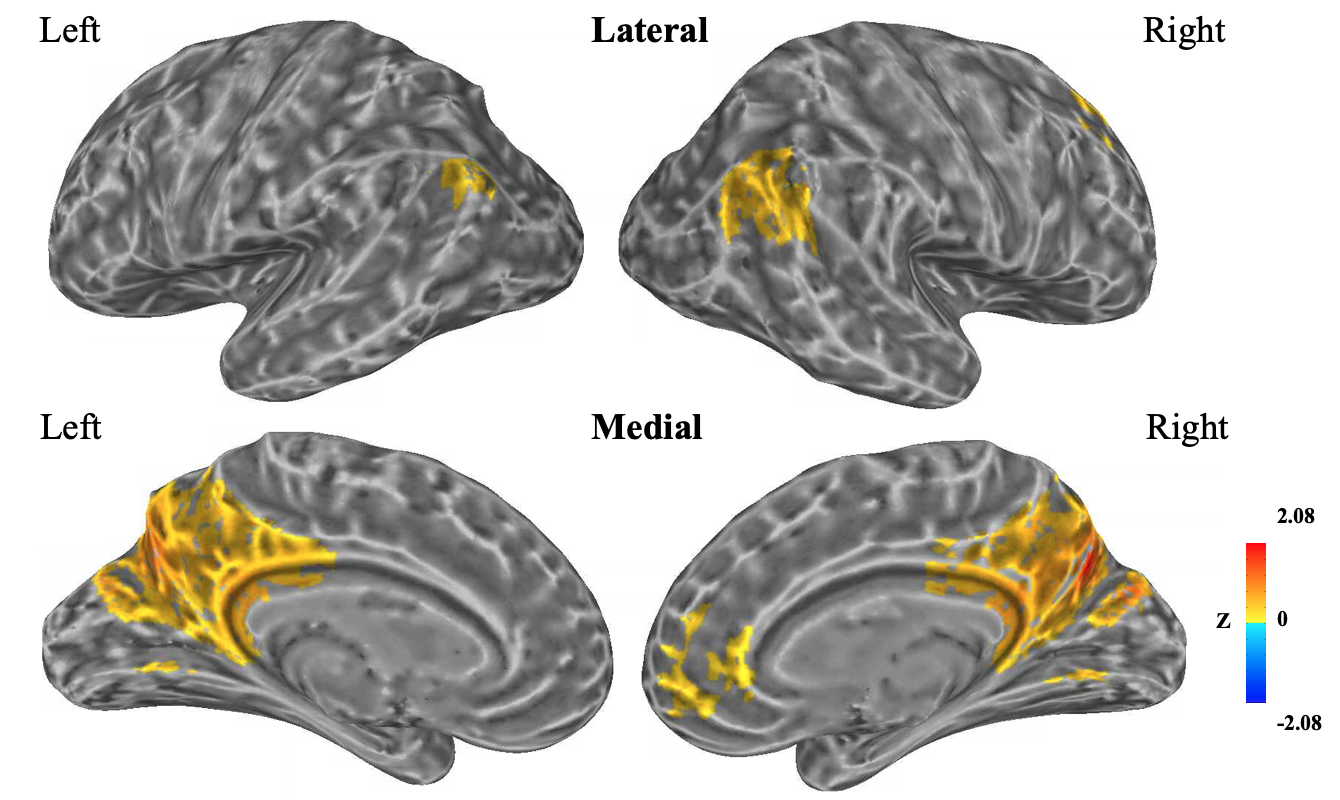


Resting-state Network 6 defined by using Cluster 6 as a seed region. The correlation coefficients between the average time-series data of the seed region with every other voxel were calculated and transformed to z scores. A *t*-test identified significant regions (*p* < 1x10^-44^, *FDR* < 3x10^-16^) that defined a resting-state network. Colors represent the value of z scores within the defined resting-state network.

**Fig S9. Resting-state Network 7**


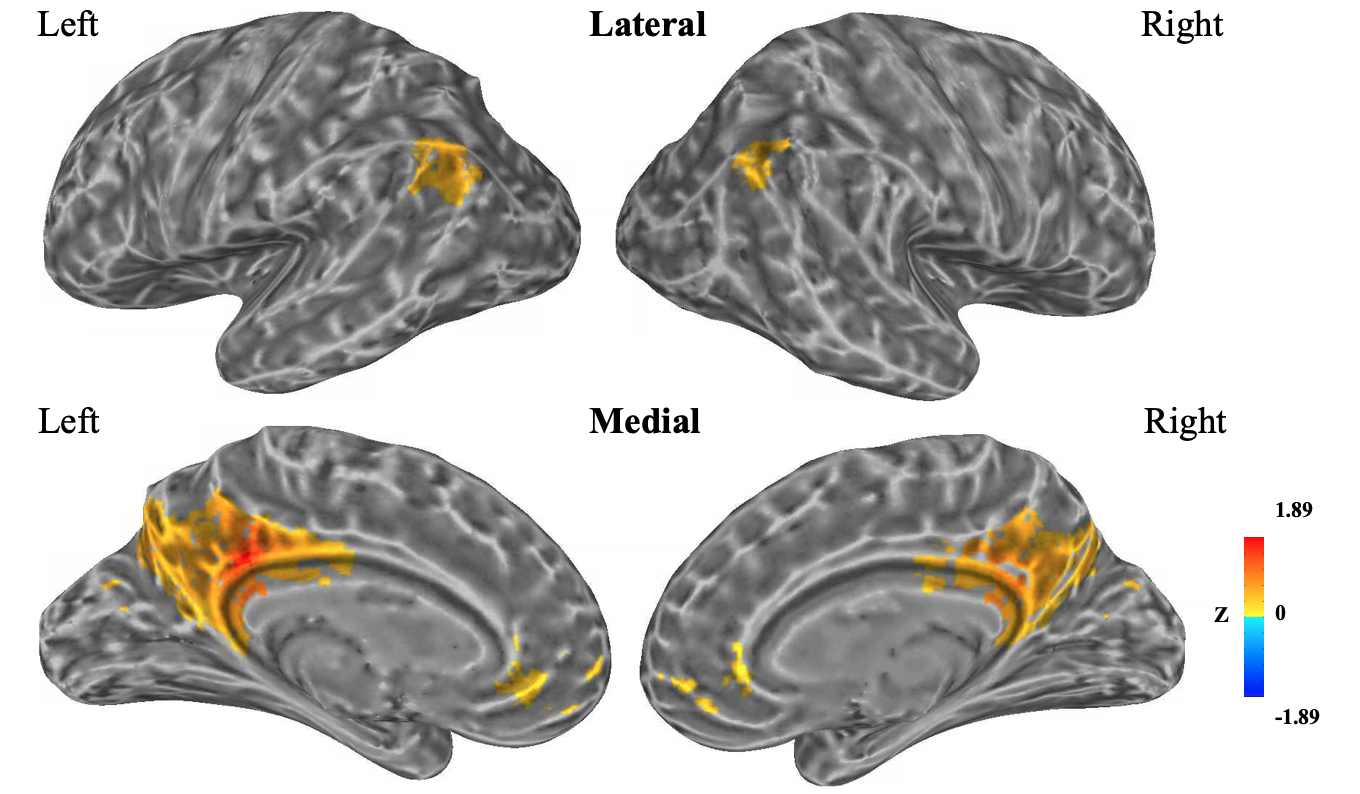


Resting-state Network 7 defined by using Cluster 7 as a seed region. The correlation coefficients between the average time-series data of the seed region with every other voxel were calculated and transformed to z scores. A *t*-test identified significant regions (*p* < 1x10^-44^, *FDR* < 3x10^-16^) that defined a resting-state network. Colors represent the value of z scores within the defined resting-state network.

**References**

Calkins, M. E., Moore, T. M., Merikangas, K. R., Burstein, M., Satterthwaite, T. D., Bilker, W. B., . . . Mentch, F. (2014). The psychosis spectrum in a young US community sample: findings from the Philadelphia Neurodevelopmental Cohort. *World Psychiatry, 13*(3), 296-305.

Cox, R. W. (1996). AFNI: software for analysis and visualization of functional magnetic resonance neuroimages. *Computers and Biomedical research, 29*(3), 162-173.

Dale, A. M., Fischl, B., & Sereno, M. I. (1999). Cortical surface-based analysis: I. Segmentation and surface reconstruction. *Neuroimage, 9*(2), 179-194.

Desikan, R. S., Ségonne, F., Fischl, B., Quinn, B. T., Dickerson, B. C., Blacker, D., . . . Hyman, B. T. (2006). An automated labeling system for subdividing the human cerebral cortex on MRI scans into gyral based regions of interest. *Neuroimage, 31*(3), 968-980.

Jo, H. J., Saad, Z. S., Simmons, W. K., Milbury, L. A., & Cox, R. W. (2010). Mapping sources of correlation in resting state FMRI, with artifact detection and removal. *Neuroimage, 52*(2), 571-582.

Satterthwaite, T. D., Connolly, J. J., Ruparel, K., Calkins, M. E., Jackson, C., Elliott, M. A., . . . Behr, M. (2016). The Philadelphia Neurodevelopmental Cohort: A publicly available resource for the study of normal and abnormal brain development in youth. *Neuroimage, 124*, 1115-1119.

Scheinost, D., Benjamin, J., Lacadie, C., Vohr, B., Schneider, K. C., Ment, L. R., . . . Constable, R. T. (2012). The intrinsic connectivity distribution: a novel contrast measure reflecting voxel level functional connectivity. *Neuroimage, 62*(3), 1510-1519.

Thomas Yeo, B., Krienen, F. M., Sepulcre, J., Sabuncu, M. R., Lashkari, D., Hollinshead, M., . . . Polimeni, J. R. (2011). The organization of the human cerebral cortex estimated by intrinsic functional connectivity. *Journal of neurophysiology, 106*(3), 1125-1165.

Xia, M., Wang, J., & He, Y. (2013). BrainNet Viewer: a network visualization tool for human brain connectomics. *PloS one, 8*(7), e68910.
